# Animal welfare in Latin America: Trends and characteristics of scientific publications

**DOI:** 10.3389/fvets.2022.1030454

**Published:** 2022-11-18

**Authors:** Carmen Gallo, Lorena Véjar, Francisco Galindo, Stella M. Huertas, Tamara Tadich

**Affiliations:** ^1^Instituto de Ciencia Animal, Facultad de Ciencias Veterinarias, Universidad Austral de Chile, Valdivia, Chile; ^2^WOAH World Organisation for Animal Health Collaborating Centre for Animal Welfare and Sustainable Livestock Systems—Chile-Uruguay-México, Valdivia, Chile; ^3^Escuela de Graduados, Facultad de Ciencias Veterinarias, Universidad Austral de Chile, Valdivia, Chile; ^4^Facultad de Medicina Veterinaria y Zootecnia, Universidad Nacional Autónoma de México, Ciudad de México, Mexico; ^5^Facultad de Veterinaria, Universidad de la República, Montevideo, Uruguay

**Keywords:** animal welfare, animal behavior, scientific publications, research, farm animals, sustainability, Latin America

## Abstract

The present study constitutes a review of the scientific articles about animal welfare in terrestrial farmed animals, published in 19 countries of Latin America. The main objectives were to quantify and characterize articles produced between 1992 and 2021 in farm animals' welfare using “Web of Science [v.5.32]” and “CAB Abstracts” databases. A total of 663 articles were found for the period analyzed, which were mainly in English (87%). The countries with the most publications were Brazil (43%), México (25%), Chile (12%), Uruguay (10%), Colombia (4%) and Argentina (2%). Cattle was the farm species most considered in the publications (41%), and the studies addressed mostly the on-farm production stage (76%). There was a rapid increase in the number of articles published in the last 15 years, accounting for 95% of the publications. This could be related to the publication of welfare standards by the World Organization for Animal Health (WOAH) since 2005, the creation of the Collaborating Center for Animal Welfare and Sustainable Livestock Systems—Chile-Uruguay-México in 2009, a Regional Strategy of Animal Welfare prompted by the WOAH in 2012 and the inclusion of animal welfare in the veterinary curriculum. The fact that most articles were in English shows that Latin American researchers have somehow overcome the challenge of publishing in a non-native language and their research can be read/cited worldwide. However considerable gaps in scientific productivity were identified in comparison to European and North American countries. Scientific research concerning the livestock industry in Latin America faces new challenges arising from the need to move toward more sustainable production systems within the One Welfare and One Health frame.

## Introduction

Animal Welfare (AW) has become an increasingly important sociocultural, scientific, political, commercial and ethical issue of debate worldwide. The focus on the welfare of farm animals has not only affected intensive livestock production systems in various species, due to the restrictive conditions in which animals are kept and the husbandry practices they are submitted to increase productivity (1–3). AW also addresses other stressful stages for production animals that are of much public concern, like transport, marketing and pre-slaughter handling in general (4). Public concern is making the livestock industry move toward more AW friendly production and handling systems that must consider, animals' behavioral needs, sustainability, traceability and ethical quality of products of animal origin (5, 6).

Scientific research has played a fundamental role in detecting critical points for the welfare of farm animals (7). The role of scientists, veterinarians and other professionals dealing with livestock production has also been crucial for scientific progress, education and legislation on these issues (7, 8). In accordance with the One Health-One Welfare framework (9) that the World Organization for Animal Health (WOAH) is applying, results have shown that the need for a more humanitarian animal production should not be seen as a barrier or threat against livestock production systems but instead as an opportunity to achieve a more sustainable livestock production (5). By improving the health and productivity of animals the quantity and quality of animal products for the consumers may also increase (1, 4, 10).

The WOAH published the first AW standards/norms in 2005, and these have been further developed continuously up to present (11). The Region of the Americas of the WOAH has 31 member countries with a wide variety of food-producing animal species and husbandry systems (12). The member countries include USA and Canada, which are among the countries with the highest scientific productivity in AW (13, 14). However, by 2006 only a few Latin American countries had a system that could finance AW research and publications on the issue (15). In order to promote AW, enhance research under local conditions and also help implement the WOAH norms in this diverse region, a Collaborating Center for Animal Welfare and Sustainable Livestock Systems Chile-Uruguay-México (https://www.woah.org/es/que-ofrecemos/red-de-expertos/centros-colaboradores/#ui-id-3) was created in 2009 (16). Further on the Regional WOAH Office for the Americas published in 2012 a Regional AW Strategy (17) that was adopted by all member countries to enhance the implementation of AW norms. At the same time, this strategy aims to promote education and applied research in AW, according to the particular regional production conditions, in order to back new legislation and improve the welfare of production animals (17). In 2015, Glass et al. (18) determined the level of awareness and implementation of the American Regional Strategy. These authors reported the existence of working groups in AW in several countries, frequent organization of seminars and other training events, production of manuals of good practices in different species and other extension activities promoting AW, but the general implementation of the AW strategy was considered to be still in an initial phase. There has been an increasing development of new laws and regulations regarding animal protection in Latin American countries since the publication of the first AW standards in 2005 (10, 18).

The development of animal welfare science in Latin America has varied greatly from region to region and scientific research is limited to a few groups (12, 16). Scientific productivity is still considered to be low in Latin America compared to other regions like North America and the European Union. The published articles worldwide on AW and related areas, according to ISI Web of Knowledge and until 2016 (14), came mainly from the United States (33.48%), followed by UK, Germany and Canada; Latin American countries (Spanish and Portuguese speaking) contributed altogether with only 7.44% of all publications, with Brazil leading the list (4.47%). According to a more recent study by Freire and Nicol (13) the USA, UK and Germany have published most of the AW scientific articles in the last 30 years (period analyzed up to 2017), and Latin American countries are not mentioned because they hardly contributed to the total. None of the above-mentioned studies **analyzed** publications in terms of farm animals specifically, the type of species, stages of production or animal products that had been included in the studies.

It appears that Latin American countries have been developing new laws, local research and increasingly applying welfare standards that enhance the welfare of production animals (10), however there has been no quantitative measure of the possible progress in terms of scientific publications. In order to highlight trends in regional research in the area of farmed animals' welfare and get an overview of the scientific productivity, the objective of the present study was to determine the quantity of publications produced in total and per country on the welfare of terrestrial farmed animals in Latin America from 1992 to 2021, as well as identify the animal species and stages of production that have been considered so far in those publications.

## Materials and methods

The methodology used in this study considered the following steps:

### Definition of literature search strategies

Keywords (within the title, summary/abstract and author key words) that were related to “animal welfare” or “animal behavior” in the area of “terrestrial production animals” corresponding to “Latin America” were selected. The search covered the years 1992 to 2021 in the CAB Abstracts (CAB) and Web of Science Core Collection (WoS) databases of the virtual library system of the Universidad Austral de Chile accessed *via* the FortClient programme. These databases were chosen because WoS had been used before in similar reviews on animal welfare publications (13, 14) and is considered worldwide an important database for scientific articles; CAB database was included because it has more journals indexed that accept articles in Spanish or Portuguese. The search and selected keywords were written according to the following strategy using Boolean search terms (AND, OR, ^*^, “, $):

- CAB Abstract (animal^*^ welfare^*^ OR animal^*^ behav^*^) AND (farm^*^ animal^*^ OR producti^*^ animal^*^ OR animal^*^ producti^*^ system^*^ OR transport^*^ OR stress^*^ OR pain^*^ OR stunn^*^ OR bruis^*^ OR handl^*^ OR slaughter^*^).
- WoS (Web of Science) (animal welfare OR animal behav^*^) AND (farm^*^ animal^*^ OR producti^*^ animal^*^ OR animal^*^ producti^*^ system^*^ OR transport^*^ OR stress^*^ OR pain^*^ OR stunn^*^ OR bruis^*^ OR handl^*^ OR slaughter^*^).

### Article inclusion/exclusion criteria

All types of scientific articles (original articles, short communications and bibliographic reviews) published from 1992 to 2021 were included in the search (done in June 2022), with no language filter, considering journals in the areas of veterinary sciences, animal science, environmental sciences and food science in both databases.

From the list of 31 countries that appear as members of the WOAH in the Region of Americas, the name of each of the 19 countries in which Spanish or Portuguese is the main language (Latin American) was selected and included as a filter: Argentina, Bolivia, Brazil, Chile, Colombia, Costa Rica, Cuba, Ecuador, El Salvador, Guatemala, Honduras, Mexico, Nicaragua, Panama, Paraguay, Peru, Dominican Republic, Uruguay, and Venezuela. Once this was done for both databases, the first raw result was obtained and the references of these 846 articles were saved in a folder on the desktop, using the option to extract in RIS format file offered by CAB and WoS. The Mendeley Desktop program was then used to open the RIS format files and a matrix table with all the information was built using the Microsoft Excel Office Version 2021 program. Based on this selection, 143 publications were manually eliminated, because abstract revealed that the study did not actually correspond to the animal welfare or animal behavior areas (i.e., were only on productive traits), still referred to non-production animals (companion, sports, laboratory or zoo animals) or non-terrestrial species (fish and other aquatic species). Of the remaining 703 articles, most (*n* = 507) were found through WoS, and less through CAB (*n* = 196). Finally, 40 articles that were duplicated because they appeared both in WoS and CAB, were also eliminated. The resulting 663 articles (WoS plus CAB) were then manually categorized considering the following variables of interest:

Authors: first author.

Title: title of the article.

Journal: title of the journal in which the article was published and language of publications.

Year of publication: the year of each publication as appearing in the journal was registered.

Country of origin: the country of the first author was used; if the first author was not from Latin America as stated by institution of origin, then the country where the study was undertaken was used.

Species: cattle (beef, dairy, purpose not specified), sheep, goats, sheep and goats, poultry (layers, broilers, other), ruminants (in general, species not specified), pigs, equids (only if abstracts revealed a relation with production, farm work or slaughter, not sports), buffalos, South American camelids, rabbits, quails, chinchillas, guinea pigs, guinea fowl, wild boar, livestock in general (studies which refer to production animals in broad terms, without specifying any), surveys to people (farmers, transporters, slaughterhouse operators, consumers/public in general, students, veterinarians).

Production stage: The articles were categorized according to the analyzed/studied productive stage in the following groups: on-farm, during transport of livestock (loading, journey, unloading), pre-slaughter (when transport and slaughter were dealt with as one item), slaughter of livestock, livestock markets, other (surveys to people or general studies throughout all production stages). Further on, within the on-farm stage, articles were sub-classified according to its contents in: articles on AW and feeding/grazing behavior, nutrition and productive parameters; articles on AW and the environment (i.e., climate and housing systems, silvopastoral systems, thermal stress); articles dealing with stress, behavioral and physiological indicators of welfare; articles on AW and reproductive handling/techniques; articles concerning the human-animal relationship and handling/moving animals; articles on specific health issues in relation to AW; articles on painful husbandry practices.

### Statistical analysis

Using the information collected in Microsoft Excel, tables were created from it to automatically count the information according to each variable. Descriptive statistics (numbers or percentages) were used and results are presented in graphs.

## Results

**Figure 1:**
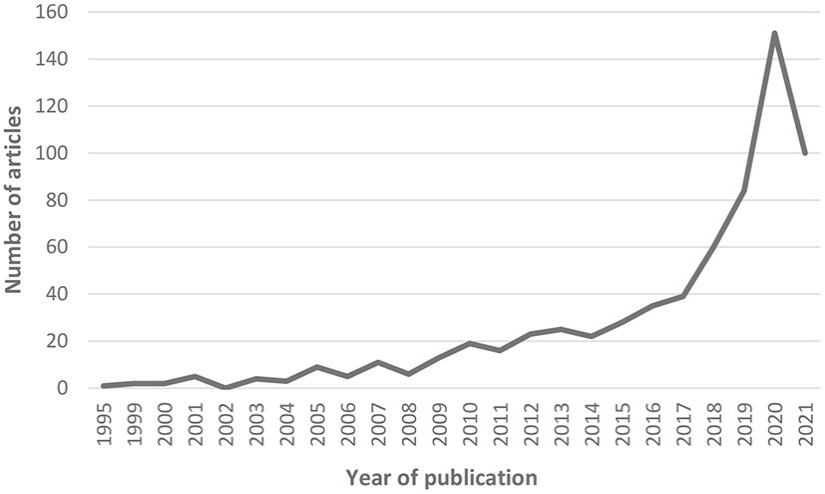
Publications on farm animal welfare in 19 countries of Latin America between 1992 and 2021 according to CAB and WoS data bases (*n* = 663).

A total of 663 published articles on farmed animals' welfare (FAW) were found for the 19 countries of Latin America between 1992 and 2021, considering the search through both databases. Regarding the distribution of the publications during the period analyzed, the earliest publication found was from 1995 by Caballero et al. in CAB (19) and there was an increase during time until 2020. A rapid increase in the total number of articles can be observed between years 2017 and 2020, where a peak of 151 articles was reached, whereas a decrease was observed in 2021 (Figure 1). Comparing the first 15-year period analyzed (1992–2006) and the last 15 years (2007–2021), 95% of all the publications was found in the latter period.

**Figure 2:**
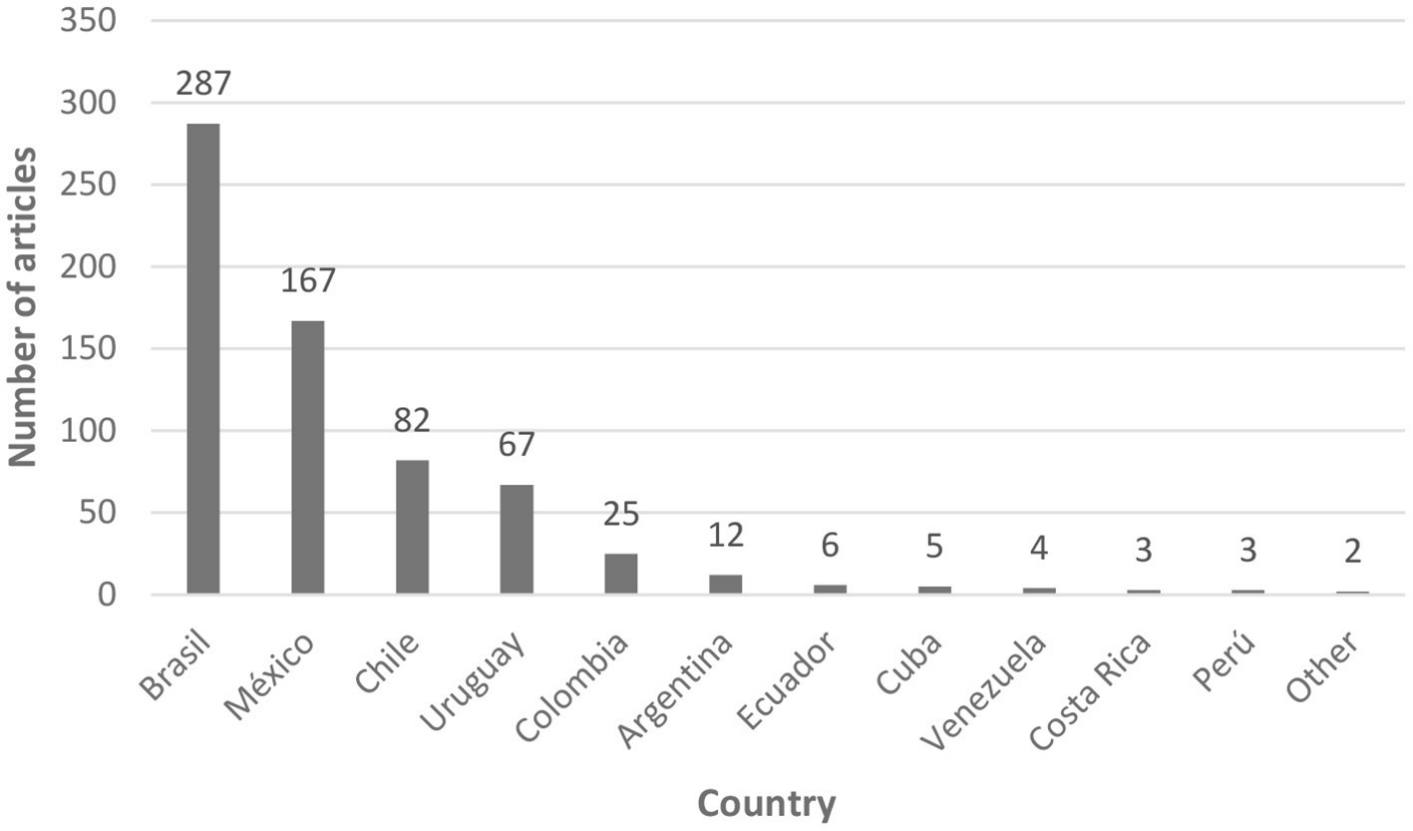
Latin American countries where scientific publications on farm animal welfare were produced between 1992 and 2021 (*n* = 663).

Considering the total of articles found (663), the countries with most publications on FAW during the period analyzed were Brazil (43%), México (25%), Chile (12%), Uruguay (10%), Colombia (4%), and Argentina (2%) (Figure 2). Ecuador, Costa Rica, Venezuela, Cuba and Perú (classified as “Others” in Figure 2) showed few publications that were also recent (2016–2018). No publications associated to FAW were found in Bolivia, El Salvador, Guatemala, Honduras, Nicaragua, Panamá, Paraguay, and Dominican Republic.

**Figure 3:**
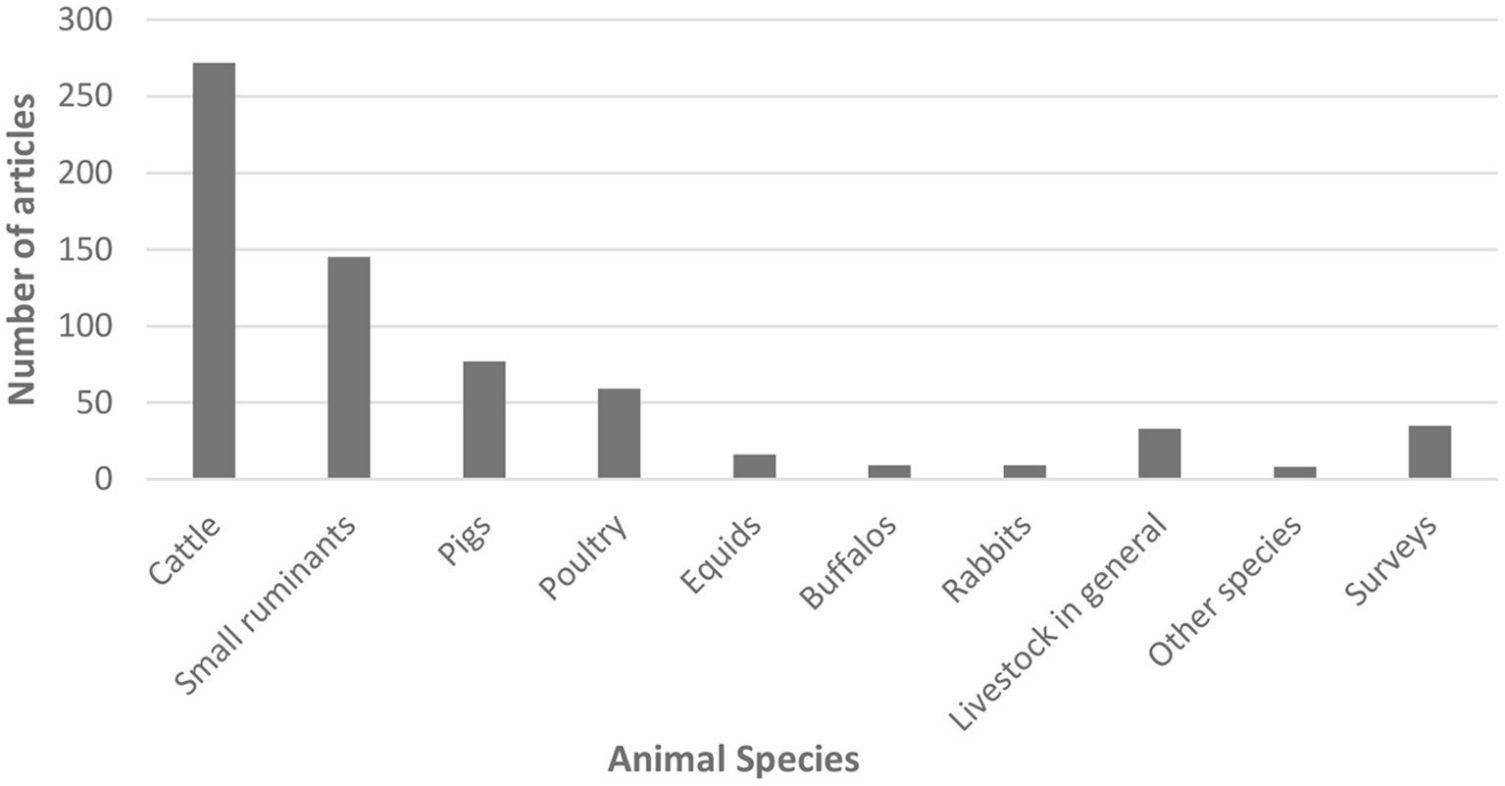
Animal species considered in the publications on farm animal welfare in Latin American countries between 1992 and 2021 (*n* = 663).

Figure 3 shows that publications on FAW in Latin America have dealt mainly with cattle (41%) and within these, more with beef (22%) than dairy cattle (19%). Studies on small ruminants were also common (22% including sheep, goats and South American camelids). Studies on pigs (12%) and poultry (9%, including broilers and layers) were less common. Among less conventional farm animal species, classified as “other species” (1%), there were articles on quails, wild boars, chinchillas, guinea fowl and guinea pigs. Five percent of articles dealt with surveys to people at different stages of production/education, aiming at their perception/appreciation/attitudes toward animal welfare.

**Figure 4:**
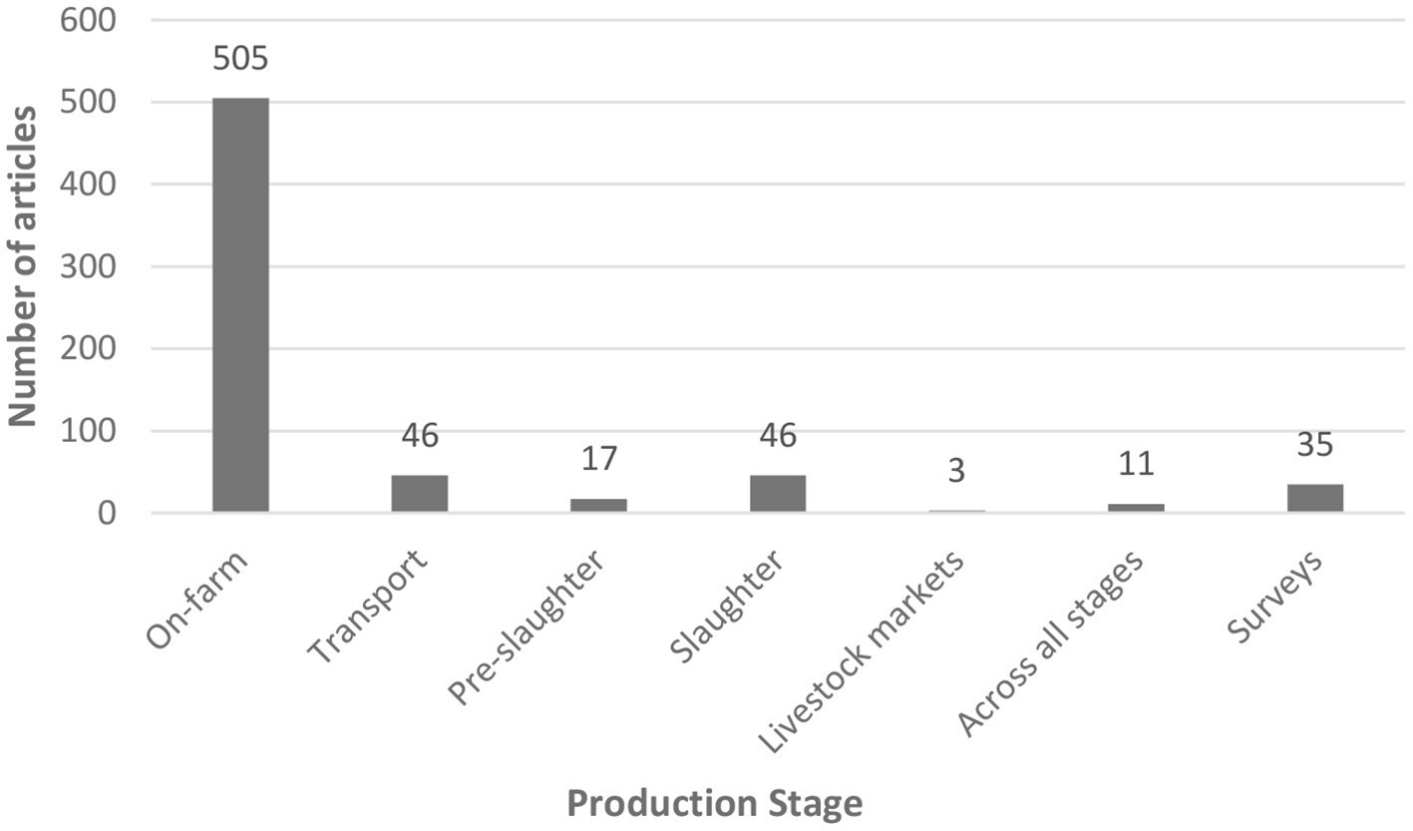
Production stages considered in the publications on farm animal welfare in Latin American countries between 1992 and 2021 (*n* = 663).

When categorizing by stage within the production chain, the on-farm stage was the most considered, covering 76% of the articles (Figure 4). Within the on-farm stage, articles on the relationship between AW and feeding/grazing behavior, nutrition and productive parameters, were the most common (28%), followed by those on AW and the environment (i.e., climate and housing systems, silvopastoral systems, thermal stress, and 19%). Articles dealing with stress, behavioral and physiological indicators of welfare (15%) and those referring to AW and reproductive handling/techniques (12.5%) were also frequent. Articles concerning the human-animal relationship and handling/moving animals (7%), specific health issues in relation to AW (6%) and painful husbandry practices (4%) were less common. Articles dealing with the transport, pre-slaughter and slaughter stages, represented altogether 16% and covered mainly issues related to transport conditions, stunning procedures and meat quality (mainly bruises, carcass pH). Studies referring to livestock markets were uncommon. The category “across all stages” included the general studies on livestock covering the whole production chain.

The articles on FAW were published in a total of 155 journals. Most journals (119) were found to accept articles in English only, whereas a few (36) accept papers in Spanish and/or Portuguese (mostly English and Spanish, or English and Portuguese). Of the 663 articles found, 576 (87%) were published in English, whereas only 87 (13%) were published in Spanish/Portuguese. The journals where most articles on FAW were found were Animals (*n* = 47), Applied Animal Behavior Science (*n* = 31), Tropical Animal Health and Production (*n* = 31), Animal Production Science (*n* = 26), Livestock Science (*n* = 24), Animal (*n* = 19), Journal of Animal Behavior and Biometeorology (*n* = 19), Austral Journal of Veterinary Science (*n* = 18), Journal of Dairy Science (*n* = 13), and with 12 articles each were Semina:Ciencias Agrarias (Londrina) Brazil, Brazilian Journal Of Animal Science, Meat Science, Journal of Veterinary Behavior: Clinical Applications and Research, and Ciencia Rural, Brazil.

## Discussion

This is a first and preliminary study giving an overview of the number and characteristics of the scientific articles on the welfare of terrestrial farm animals published in Latin America, covering a period of 30 years (between 1992 and 2021). The articles were analyzed in terms of number and year of publication, country of origin, animal species involved and production stages considered in the studies, as well as the journals and language of the publication, which will be discussed in the next sections.

### Number of articles during the period analyzed

Our results agree with those of earlier bibliographic reviews (13, 14), showing that the productivity of scientific articles on AW in Latin American countries (*n* = 663) is in general low compared to that of countries from North America and the European Union. Freire and Nicol (13) collected their data worldwide from the WoS, core collection-science citation index expanded (SCIEXPANDED 1968–2017), all languages and all types of documents and found between 10,349 and 15,614 publications on AW in general; however, they did not provide any numbers for publications originating specifically in Latin American countries. Mota-Rojas et al. (14) searched for publications in AW in general in Latin America plus Spain (“Iberoamerica”), using the Journal Citation Reports database in the Web of Knowledge and found 2,537 publications from Brazil, 669 from Mexico and 210 from Chile. In the present study we found 663 articles through the WoS and CAB databases and collected publications on the welfare of terrestrial farm animals only, greatly reducing the scope by excluding all publications on AW in aquatic animals, wild and zoo animals, sport horses, pets and laboratory animals. Considering that there is always a risk of bias in the selection of the key words and search words in this type of studies as indicated by Freire and Nicol (13), some articles might not have been found with the search words used, which means that there is a risk that publications may be underestimated in our study. For instance, in the case of Chile and Uruguay, and perhaps other countries, the words “animal welfare” and “welfare” were not included in many of the older publications on AW, because there was some reticence from financing institutions to finance studies and research projects dealing directly with the issue, hence the authors avoided using the term specifically. More than actual numbers this study gives a preliminary insight on the trends of scientific articles on farm animal welfare (FAW) in Latin America.

Our study shows a large increase in the number of publications on FAW in Latin America throughout the time period analyzed, which coincides with the results of Freire and Nicol (13) for AW publications in general worldwide. However, in the case of Latin America, the increase is more recent, finding 95% of all articles published between 2007 and 2021, which indicates that animal welfare and behavior issues started developing in Latin America much later than in Europe and North America. The increasing interest in FAW in Latin America could be due to a worldwide trend observed toward animal welfare issues (20). This includes consumer and social pressure in general (21–23), and also the work of WOAH in publishing the AW standards (norms) for the first time in 2005 and promoting their implementation in member countries (11, 17). The need to include AW as an issue in international trade was also important in Latin America, where several of the main beef exporters are located (24, 25). For example, Brazil accounts for 23.5% of the world beef exports, Argentina 7.58%, Uruguay 3.81% and México 3.17% (26).

Another driver of research and publications in animal welfare could be related to education and regulation politics in the WOAH and the Latin American member countries. The implementation of animal welfare standards by Member Countries of the WOAH was determined in 2009 through a survey in 172 member countries (27). According to that survey, 66% of those countries identified veterinarians as the main responsible people behind the implementation of the standards and the development of legislation on AW in all countries. If veterinarians play a fundamental role in the study and research of AW and the implementation of politics in each country, then another important factor for the increase in publications may be that AW has been included by WOAH within the core curriculum in veterinary education (28). AW has been increasingly included as a compulsory subject in the curriculum of veterinarians and other professionals working in the livestock industry in Latin America since 2013 (29–31). In 2008 a 63% of the veterinary schools had at least one AW course included in the curriculum (32) whereas in 2016, in a sample of 100 out of the around 400 existing veterinary schools, this was true for 98% of them (31). The effects of the Regional AW Strategy of the Americas were only in an initial phase in 2015 (18), but it has certainly promoted further development of regulations in AW in many Latin American countries (10). Finally, due to the present economic and political importance of the subject of AW for many Latin American countries that are exporting animal products (24, 25), research financing institutions have also been prioritizing issues related to FAW in the last years.

Speaking from the experience of the authors, it was not easy to get funding for research in AW in the 90's as it was a new subject, sometimes regarded as a passing trend and thought to be incompatible with production systems. The creation of the WOAH Collaborating Center for Animal Welfare and Sustainable Livestock Systems in this Region in 2009, has also prompted research in AW. It has disseminated results through the organization of large international conferences on AW in the three participating countries (in Chile 2009, 2018; in Uruguay 2012, 2022 and in México 2015). At these conferences young researchers from all Latin American countries have the opportunity to present their studies as well as meet colleagues and start collaborative research. The last conference gathered over 100 poster presentations and was held together with the regional International Society for Applied Ethology (ISAE) conference in 2018 (book of abstracts available at https://www.bienestaranimal.cl/wp-content/uploads/2019/07/Libro-de-Resumenes-BAISAE-2018.pdf), the most important scientific society on animal welfare science and active in Latin America since the early 90's (12, 16). Coincidently, the young researchers presenting their initial studies at our first meeting in 2009, are now heading their own research groups in AW in several Latin American countries and publishing their work.

The decrease in the number of published articles observed in 2021 could be related to the COVID-19 pandemic, but there are also other issues to be considered for the future of research and publications. An important factor is the large increase in the publication costs (APCs) imposed by most journals, which are difficult to be financed by many Latin American institutions and researchers, since they are usually higher than a researcher's monthly salary.

### Main countries of origin of the publications on FAW

The main countries of origin of the articles on FAW were Brazil and México, which agrees with the findings of Mota-Rojas et al. (14) in his search for articles on animal welfare in Iberamerica. Freire and Nicol (13) also mention Brazil as the only visible Latin American country in their study of the scientific publications on AW worldwide, although they also mention that these articles have few citations. According to our study, Brazil, México, Chile, Colombia, Uruguay, and Argentina produced 96% of all articles on FAW. The leadership of Brazil in research and publications related to the livestock industry, in general, is probably due to its large geography within America, holding a similarly high human and cattle population (around 200 million each), and being the main meat exporter of the world (33). Besides beef exports, Brazil is also a main exporter of broiler and pork meat (34). México is also a large country in terms of human population and has a considerable cattle population (33 million) with a wide variety of husbandry systems. Another interesting factor may be that both countries also have many local journals that publish research findings in English and are WoS indexed, such as the Brazilian Journal of Animal Science, Ciencia Rural (Brazil), Revista Mexicana de Ciencias Pecuarias and Veterinaria México.

The WOAH Collaborating Center of AW and Sustainable Livestock Systems Chile-Uruguay-México has the objective of promoting AW in the region, hence it is not surprising that these countries were productive in terms of publications. There are groups of researchers on AW in each of these countries, which have networks or connections with researchers from most other Latin American countries (35). Accordingly, Universidad Nacional Autónoma de México, Universidad Austral de Chile and Universidad de la República in Uruguay, as part of the Collaborating Center have developed diverse strategies to promote the application of AW regulations and integrate AW within the production systems in Latin America (12, 16, 18, 35–37).

Only a few publications (appearing since 2016) were found in Cuba, Ecuador, Venezuela, Perú and Costa Rica, and none originated from Bolivia, El Salvador, Guatemala, Honduras, Nicaragua, Panamá, Paraguay and the Dominican Republic. This could be explained by the fact that several of these countries base their economies on activities different from livestock production (38, 39). However, Paraguay has a large cattle population and is a meat exporter and Bolivia has a similar situation. These countries may lack support for research from financing institutions, which prevents the development of research that could enhance their productive standards and improve the ethical quality of their products. According to the World Bank (40) the percentage of the gross domestic product (GDP) that Latin American countries invest in science and technology is still low, especially if compared to more developed countries. For example, Brazil invests the highest percentage in science and technology with 1.21%, followed by Uruguay (0.48%), Argentina (0.46%), Chile (0.34%), and México (0.3%), but countries as Perú, Bolivia, Paraguay are around 0.1% and Nicaragua only invests 0.03%. These percentages are much lower than the over 3% invested by the USA and Germany, and over 1.5% by Canada and the UK (40). The demands of countries from the European Union have encouraged countries like Brazil, Chile, Uruguay and Argentina to produce under higher welfare standards, and this could have been a driver for more research and then using evidence-based results for supporting changes in livestock handling and within the legislation (10, 25, 38, 41).

### Characteristics of the publications on FAW: Species and stages of production

In terms of the characteristics of the publications and their contents, we found that these dealt mainly with cattle (41% of the articles) during the on-farm stage. Cattle is a farm species with high population in most Latin American countries (39) and is also the most considered species worldwide in AW studies (13, 42). Our results show that the articles on FAW in Latin America dealt more with beef (22%) than dairy cattle (19%). This coincides with the fact that in Latin America we have several countries that are large beef producers and exporters, and therefore the interest in the welfare of farm animals and its relationship with meat quality was an initial driver for research (35, 43, 44). However, it differs from Freire and Nicol (13) who found that publications on AW worldwide dealt mainly with dairy cattle and were related to milk production and associated illnesses, such as lameness and mastitis. Recent studies in Chile and Brazil have also shown how cow welfare and productivity can be affected by lameness and mastitis (45–47) and a similar approach has been used looking at the welfare of dairy calves in relation to management, behavior and performance (48–51). Differences between studies and regions, are probably due to the fact that the dairy production systems in Europe, USA and Canada are more intensive and frequently combined with indoor housing, which often have worse welfare than extensively raised animals when we consider lack of comfort, insufficient space availability and fewer opportunities to perform natural behaviors (52). These characteristics pose a greater risk of welfare problems in more intensive systems and a greater need for research to find solutions. Although extensive production systems are generally regarded as more natural and welfare friendly, they may not provide livestock with enough shelter from inclement weather, food or water (extreme climate events), or protection from predators. This agrees with our findings on the topics most considered within the on-farm stage: 28% of the publications dealt with nutrition (feeding, grazing behavior in relation to productivity and AW) and 19% with comfort of the environment (climate, housing, thermal stress and others). Because beef and milk is produced mainly on large farms where animals are on pasture all year round, there is a growing interest in the welfare and productivity of dairy and beef cattle under heat stress and studies on the use of silvopastoral systems to mitigate heat stress and improve welfare have been undertaken recently (53–56). But the climate and the geography of Latin America is so variable, that the effects of cold and wet environments have also been considered recently in relation to welfare (57, 58).

At the beginning, Latin American publications dealt importantly with the welfare of meat producing species (cattle, sheep, pigs, broilers) which includes not only the stage of production on farm but also the transport, handling and slaughter stages (59–62). Hence earlier research focused on the relationship between AW and the quantity and quality of meat produced, which may be applied to all species producing meat for human consumption and is directly related to economic losses (43, 44, 63, 64). Several of the initial studies on long distance transport of cattle for slaughter in Chile and other countries in Latin America used productive (weight loss, carcass yield), health (mortality, lesions), stress (blood variables) and product quality (bruises and muscle pH) as AW indicators (44, 64–70). This was due mainly because countries like Brazil, Uruguay, Argentina and Chile have had the political and consumer pressure for including AW within their quality assurance schemes to be able to sell their meat to European countries, which are more demanding in terms of welfare. Today, AW has been recognized as part of the One Health/ One Welfare concept (9) and an important issue related to the development of livestock productivity and sustainability (5, 71). Although research was initially more directed toward meat quality during the preslaughter stages and considered mainly productive indicators of welfare, it could be noticed in our review that more recent studies are increasingly using behavioral indicators of welfare that express not only negative but also positive emotional states and cognition of the animals (48, 50, 54, 72–75).

Studies analyzing compliance and impact of good handling practices on farm have also been undertaken in several countries and species (52, 76–78). Results show that there is still much research and publishing to do on species like poultry (layers and broilers) and swine, which are also exported as pork meat to Europe and Asia (39). Surprisingly, there are very few studies on species that one might think are related to smaller producers and important culturally, like South-American camelids or guinea pigs. We found only two articles on camelids (79, 80) and one in guinea pigs (81).

Animal suffering due to common husbandry practices during the on-farm stage of production like tail docking, dehorning and castration in various species has been an issue of debate among farmers, practitioners and the public in general. It was interesting to find several surveys in Latin America dealing with the perception of pain in animals by farmers and veterinary professionals, as well as studies on the effects of these husbandry practices directly on the expression of pain and stress in the animals (82–88). On the other hand, the tendency of people to increasingly consume more organic products and those produced under welfare friendly systems that avoid animal suffering as much as possible is growing (3, 20, 89). Several surveys on the issue were published during the last few years on the perception of Latin American consumers (90–95). There is a growing trend for livestock products to have a certification for animal welfare either from national or international certification bodies. Cage-free and free-range egg production systems in Latin America is a field of increasing interest, however, it appears that there is still a lack of knowledge related to the AW certifications and what these mean when it comes to consumers preferring one product over another (95). A recent survey by Cornish et al. (96) revealed that there is a better understanding and acceptance of certified products by consumers when they do not only get an AW seal, but also educational information on what parameters/indicators have been used to certify them and how the specific standards have been met.

Research in Latin America has expanded, moving from a Eurocentric perspective on the type of systems and problems studied to a wider spectrum of topics that in a way are the reflection of the diversity of agroecosystems and husbandry systems in the region. Efforts are still needed to promote and support more local research and the development of efficient policies based on sound science. In this sense, the WOAH has the potential to be a driver to strengthen networking with local actors, especially producers' organizations and industry in order to promote investment for a more strategic collaborative research on animal welfare.

### Journals and language

Freire and Nicol (13) agreed on the need to close the gaps associated with language that are related to AW publications in Latin American countries. Scientific articles in Spanish or Portuguese have a reduced possibility to be read (and hence to be cited) because these will be shared mainly within Latin America and perhaps Spain and Portugal. Similarly, Sinclair et al. (97) reported that few articles on animal welfare in China have been translated into English and thus are unavailable for the global scientific community. This could create a misleading perception of a lack of interest about animal welfare in China. Our results show that 87% of the articles on FAW found in this search were published in journals that only accept articles in English. This shows that Latin American researchers have somehow overcome the difficulties of publishing in a non-native language, which used to create a significant barrier for publishing in high impact factor journals. Speaking from the experience of the authors, it is common that Latin American universities and institutions encourage their research staff to publish in English, because articles (and therefore also institutions) will get more visibility/readability and the likelihood of being cited increases. The fact that academic career is evaluated in terms of scientific productivity and impact of publications (10) and that in some universities researchers receive economic incentives for publications in high impact journals, has probably been an important driver in some Latin American countries for the noticeable increase in publications observed in the last 15 years and for publishing in English rather than in Spanish/Portuguese.

A strategy used by several of the most productive Latin American authors to facilitate publishing in English and increase productivity and readability of their articles has been to work and publish in collaboration with North American and European English-speaking researchers who work in the same fields within FAW. Although in the present study we did not quantify how many articles have been coauthored with researchers/institutes from regions outside of Latin America, some examples of these joint publications are von Keyserlingk and Hötzel (2), Gallo et al. (59–61), Huertas et al. (98), Broom et al. (99), Tadich et al. (100), Strappini et al. (101), Miranda de la Lama et al. (63). This is the result of the interaction between key international researchers in FAW, many of whom have been doctoral or master's thesis supervisors of younger Latin American researchers or have met at international conferences and then been invited to visit and speak at conferences in Latin America. This interaction between researchers from other regions has facilitated collaborative research and also publishing in English. Collaborative networking among Latin American researchers in FAW has also been successful and authors of different countries within the region were identified to be linked through co-authoring publications (29, 32, 35, 43, 74, 102–105). Further analysis should follow in order to provide quantitative data regarding the groups of researchers working in specific topics of farm animal welfare, the main authors and their connections within the region and with other regions, because this could help enhancing animal welfare development in Latin America.

## Conclusions

The number of publications on farm animal welfare in Latin America is still low compared to more developed regions of the world, however, an important increase in articles was found during the last 15 years. This could be related to the implementation of the WOAH standards for animal welfare worldwide since 2005, but also to political reasons that have included animal welfare as an issue in international trade and the consequent interest of Latin American countries to increase research in the area in order to meet certain welfare standards. In fact, the six countries (Brasil, México, Chile, Colombia, Uruguay and Argentina) that produced 96% of all articles on farm animal welfare are important meat exporters. This coincides with the fact that most publications dealt with meat production species like cattle, sheep, pigs and poultry, during the on-farm production stage.

Another driver for the increase in publications could have been the inclusion of animal welfare within the veterinary curriculum, which opened new areas of research for the students, as well as universities prompting their staff to publish in high impact journals. The fact that most of the articles on farm animal welfare in Latin America were in journals that publish in English shows that Latin American researchers have somehow overcome the language problem and their research can be read/cited worldwide. Further analysis of the publications on farm animal welfare in Latin America should include citations of the articles, as well as identifying research groups/authors and networking, in order to provide information on the impact research in this region may have worldwide.

## Author contributions

CG was responsible for general supervision and writing the first draft. LV was responsible for the search and initial analysis, TT was responsible for analysis and descriptive statistics. CG, TT, SH, and FG contributed to the writing and discussion of the manuscript in its final version. All authors contributed to the article and approved the submitted version.

## Funding

Escuela de Graduados, Facultad de Medicina Veterinaria, Universidad Austral de Chile funded the Masters Programme of LV.

## Conflict of interest

The authors declare that the research was conducted in the absence of any commercial or financial relationships that could be construed as a potential conflict of interest.

## Publisher's note

All claims expressed in this article are solely those of the authors and do not necessarily represent those of their affiliated organizations, or those of the publisher, the editors and the reviewers. Any product that may be evaluated in this article, or claim that may be made by its manufacturer, is not guaranteed or endorsed by the publisher.

## References

[1] Molento CFM Bond GB. Production and animal welfare; ethical and technical aspects of bovine production. Ciênc Vet Tróp. (2008) 11(Supl 1):36–55.

[2] Von Keyserlingk M Hötzel MJ. The ticking clock: addressing farm animal welfare in emerging countries. J Agric Environ Ethics. (2015) 28:179–95. 10.1007/s10806-014-9518-7

[3] Shields S Shapiro P Rowan A. A decade of progress towards ending the intensive confinement of farm animals in the United States. Animals. (2017) 7:40. 10.3390/ani7050040 28505141 PMC5447922

[4] Broom DM. Animal welfare: an aspect of care, sustainability, and food quality required by the public. J Vet Med Educ. (2010) 37:83–8. 10.3138/jvme.37.1.83 20378884

[5] Keeling L Tunón H Olmos Antillón G Berg C Jone M Stuard L Swanson J Wallenbec A Winckler C Blokhuis H. Animal welfare and the United Nations sustainable development goals. Front Vet Sci. (2019) 6:336. 10.3389/fvets.2019.00336 31649940 PMC6797006

[6] Olmos-Antillón G Tunón H de Oliveira D Jones M Wallenbeck A Swanson J. Animal welfare and the United Nations' sustainable development goals—broadening students' perspectives. Sustainability. (2021) 13:3328. 10.3390/su13063328

[7] Manteca X. Tendencias de la investigación científica en Bienestar Animal. In:González G Stuardo L Benavides P Villalobos P, editors. Actas del Seminario La Institucionalización del Bienestar Animal, un Requisito para su Desarrollo Normativo, Científico y Productivo. Santiago de Chile: Salvat Impresores (2004), 29–44.

[8] Ventura B Weary D Giovanetti A Von Keyserlingk M. Veterinary perspectives on cattle welfare challenges and solutions. Livest Sci. (2016) 193:95–102. 10.1016/j.livsci.2016.10.00433663825

[9] García Pinillos R Appleby MC Manteca X Scott-Park F Smith C Velarde A. One welfare-a platform for improving human and animal welfare. Vet Rec. (2016) 179:412–3. 10.1136/vr.i5470 27770094

[10] Gallo C Tadich T. Perspective from Latin America. In:Mench J, editors. Advances in Agricultural Animal Welfare. Science and Practice. Duxford: Woodhead Publishing (2017), 197–218. 10.1016/B978-0-08-101215-4.00011-0

[11] WOAH (World Organization for Animal Health). Terrestrial Code. 7, Animal Welfare. Ed. Paris: OIE (2022). Available online at: https://www.woah.org/en/what-we-do/standards/codes-and-manuals/terrestrial-code-online-access/?id=169&L=1&htmfile=titre_1.7.htm (accessed August, 2022).

[12] Galindo F Huertas SM Gallo C. Recomendaciones de la OIE para la enseñanza de la Etología y el Bienestar Animal en el contexto de la estrategia regional para las Américas. In:Taylor-Preciado JJ, editors. Inclusión de temas de Bienestar Animal en planes de estudio de Medicina Veterinaria en Latinoamérica. Guadalajara: Amateditorial S.A. (2016), 9–16.

[13] Freire R Nicol CJ. A bibliometric analysis of past and emergent trends in animal welfare. Anim Welfare. (2019) 28:465–85. 10.7120/09627286.28.4.465

[14] Mota-Rojas D Taylor-Preciado J Ramírez-Necoechea R Mora-Medina P. Bienestar Animal en Iberoamérica: seguimiento de artículos científicos. In:Taylor-Preciado JJ, editors. Inclusión de temas de Bienestar Animal en planes de estudio de Medicina Veterinaria en Latinoamérica. Guadalajara: Amateditorial S.A. (2016), 31–9.

[15] Gallo C. Animal Welfare in the Americas. In: Proceedings of Technical Items Presented to the International Committee and Regional OIE Commissions, Florianopolis, Brazil 2016. Paris: World Organization for Animal Health (2006), 159–66.

[16] Galindo F Tadich T Ungerfeld R Hotzel MJ Miguel-Pacheco J. The development of applied ethology in Latin America. In:Brown J and Seddon Y, editors. Animals and us: 50 years and more of applied ethology, ISAE 50th Anniversary, 1st Edn. Netherlands: Wageningen Academic Publishers (2016), 211–25. 10.3920/978-90-8686-828-5_10

[17] WOAH (World Organization for Animal Health). Regional Animal Welfare Strategy for the Americas. Paris: WOAH (World Organization for Animal Health) (2020). Available online at: https://rr-americas.oie.int/wp-content/uploads/2020/01/estrategia-regional-reporte-final-barbados_esp.pdf

[18] Glass E Kahn S Arroyo Kuribreña M. Awareness and implementation of the regional animal welfare strategy for the Americas: a questionnaire. Rev Sci Tech Off Int Epiz. (2015) 34:673–88. 10.20506/rst.34.3.2388 27044144

[19] Caballero C Ocampo C Sumano L. A review of the use of recombinant bovine somatotropin during conditions of heat stress in dairy cattle. Técnica Pec México. (1995) 33:168–77.

[20] Verbeke W. Stakeholder, citizen and consumer interests in farm animal welfare. Anim Welfare. (2009) 18:325–33.

[21] Santurtún Oliveros E Tapia Pérez G González Rebeles C Galindo F. Consumer attitudes and perceptions towards sustainable animal production attributes in Mexico City. Veterinaria México OA. (2012) 43:87–101. Available online at: https://www.scielo.org.mx/pdf/vetmex/v43n2/v43n2a1.pdf

[22] Lama GC Estévez-Moreno LX Sepúlveda WS Estrada-Chavero MC Rayas-Amor AA Villarroel M. Mexican consumers' perceptions and attitudes towards farm animal welfare and willingness to pay for welfare friendly meat products. Meat Sci. (2017) 125:106–13. 10.1016/j.meatsci.2016.12.001 27940228

[23] Rossi Borges JA de Faria Domingues CH Ribeiro Caldara F da Rosa NP Senger I Gomes Freire Guidolin D. Identifying the factors impacting on farmers' intention to adopt animal friendly practices. Prevent Vet Med. (2019) 170:104718. 10.1016/j.prevetmed.2019.104718 31421489

[24] Thiermann A Babcock S. Animal welfare and international trade. Rev Sci Tech Int Off Epiz. (2005) 24:747–55. 10.20506/rst.24.2.1600 16358524

[25] González G Stuardo L Benavides P Villalobos P. Actas del Seminario La Institucionalización del Bienestar Animal, un Requisito para su Desarrollo Normativo, Cientí*fico y Productivo*. Santiago de Chile: Salvat Impresores (2004), 15–6.

[26] USDA. Ranking of Countries that Export the Most Beef. Washington, DC: USDA. Available online at: https://beef2live.com/story-world-beef-exports-ranking-countries-0-106903-printversion

[27] Stafford K Mellor D. The implementation of animal welfare standards by Member Countries of the World Organisation for Animal Health (OIE): analysis of an OIE questionnaire. Rev Sci Tech Off Int Epiz. (2009) 28:1143–64. 10.20506/rst.28.3.1959 20462173

[28] WOAH World Organisation for Animal Health. (OIE [Office International des Épizooties]) Veterinary Education Core Curriculum OIE Guidelines. Paris: WOAH (World Organization for Animal Health) (2013).

[29] Mota-Rojas D Orihuela A Strappini A Cajiao M Agüera E Mora-Medina P. Teaching animal welfare in veterinary schools in Latin America. Int J Vet Sci Med. (2018) 6:131–40. 10.1016/j.ijvsm.2018.07.003 30564587 PMC6286393

[30] Hewson C Baranyiová E Broom DM Cockram MS Galindo F. Approaches to teaching animal welfare at thirteen veterinary schools in Europe, North America and South America. J Vet Med Educ. (2005) 32:422–37. 10.3138/jvme.32.4.422 16421823

[31] Taylor-Preciado JJ. Inclusión de temas de bienestar animal en planes de estudio de Medicina Veterinaria en Latinoamérica. In: Amat, editorial SA, editor. Asociación Panamericana de Ciencias Veterinarias (PANVET), Federación Panamericana de Facultades y Escuelas de Ciencias Veterinarias. Guadalajara: FAO y Universidad de Guadalajara (2016), 153.

[32] Tadich N Molento C Gallo C. Teaching animal welfare in some veterinary schools in Latin America. J Vet Med Educ. (2010) 37:69–73. 10.3138/jvme.37.1.69 20378882

[33] IBGE (Instituto Brasileiro de Geografia e Estatística). Pesquisa Trimestral do Abate de Animais. Brazil: IBGE (2021). Available online at: https://www.ibge.gov.br/estatisticas/economicas/agricultura-epecuaria/9203-pesquisas-trimestrais-do-abate-de-animais.html?=&t=o-que-e (accessed September 28, 2021).

[34] Juárez C,. Brasil, líder en la exportación de carne bovina y de pollo. Sterling, VA: The Food Tech (2021). Available online at: https://thefoodtech.com/industria-alimentaria-hoy/brasil-lider-en-la-exportacion-de-carne-bovina-y-de-pollo (accessed September 12, 2021).

[35] Huertas S Gallo C Galindo F. Drivers of animal welfare policy in the Americas. Rev Sci Tech Off Int Epiz. (2014) 33:67–76. 10.20506/rst.33.1.2264 25000778

[36] Rojas H Stuardo L Benavides D. Animal health policies and practices in the Americas: preliminary study. Rev Sci Tech Int Off Épiz. (2005), 24:549–65. 10.20506/rst.24.2.1589 16358507

[37] Tadich T Stuardo L. Strategies for improving the welfare of working equids in the Americas: a Chilean example. Rev Sci Tech Int Off Epiz. (2014) 33:203–11. 10.20506/rst.33.1.2271 25000793

[38] Labraga J. Exportaciones de Carne Bovina del MERCOSUR: Una Cuantificación de Los Efectos Comerciales de Medidas Sanitarias Nuevas y Tradicionales. Julio: Instituto para la Integración de América Latina y el Caribe (INTAL) IDB-TN-1046 (2016). Available online at: https://publications.iadb.org/publications/spanish/document/Exportaciones-de-carne-bovina-del-MERCOSUR-Una-cuantificaci%C3%B3n-de-los-efectos-comerciales-de-medidas-sanitarias-nuevas-y-tradicionales.pdf

[39] FAO. Situación del Mercado: Carne. Rome: FAO (2017). Available online at: https://www.fao.org/3/BT089s/BT089s.pdf (accessed June 4, 2021).

[40] World Bank. Research and Development Expenditure (% of GDP). Washington, DC: World Bank (2022). Available online at: https://data.worldbank.org/indicator/GB.XPD.RSDV.GD.ZS (accessed July 7, 2022).

[41] Bowles D Paskin R Gutiérrez M Kasterine A. Animal welfare and developing countries: opportunities for trade in high-welfare products from developing countries. Rev Sci Tech Off Int Epiz. (2005) 24:783–90. 10.20506/rst.24.2.1610 16358527

[42] Von Keyserlingk M Weary D. A 100-year review: animal welfare in the journal of dairy science—the first 100 years. J Dairy Sci. (2017) 100:10432–44. 10.3168/jds.2017-13298 29153174

[43] Paranhos Da Costa M Huertas S Gallo C Dalla Costa O. Strategies to promote farm animal welfare in Latin America and their effects on carcass and meat quality traits. Meat Sci. (2012) 92:221–6. 10.1016/j.meatsci.2012.03.005 22503613

[44] Gallo CB Huertas SM. Main animal welfare problems in ruminant livestock during preslaughter operations: a South-American view. Animal. (2016) 10:342–8. 10.1017/S1751731115001597 26251114

[45] Tadich N Hettich E Schaik G. Prevalence of lameness in cows from 50 dairy herds in southern Chile. Arch Med Vet. (2005) 37:29–36. 10.4067/S0301-732X2005000100005

[46] Green L Borkert J Monti G Tadich N. Associations between lesion-specific lameness and the milk yield of 1,635 dairy cows from seven herds in the Xth region of Chile and implications for management of lame dairy cows worldwide. Anim Welfare. (2010) 19:419–27.

[47] Bran J Costa J Keyserlingk M Hötzel M. Factors associated with lameness prevalence in lactating cows housed in freestall and compost-bedded pack dairy farms in southern Brazil. Prev Vet Med. (2019) 172:104773. 10.1016/j.prevetmed.2019.104773 31563110

[48] Hoetzel MJ Ungerfeld R Quintans G. Behavioral responses of 6-month-old beef calves prevented from suckling: influence of dam's milk yield. Anim Prod Sci. (2010) 50:909–15. 10.1071/AN09136

[49] Hoetzel MJ Longo C Balcao LF Cardoso CS Costa JHC. A survey of management practices that influence performance and welfare of dairy calves reared in southern Brazil. PLoS ONE. (2014) 9:e114995. 10.1371/journal.pone.0114995 25506692 PMC4266638

[50] Calderón-Amor JA Beaver A von Keyserlingk MAG Gallo C. Calf- and herd-level factors associated with dairy-calf reactivity. J Dairy Sci. (2020) 103:4606–17. 10.3168/jds.2019-16878 32147267

[51] Calderón-Amor J Gallo C. Dairy calf welfare and factors associated with diarrhea and respiratory disease among Chilean dairy farms. Animals. (2020) 10:1115. 10.3390/ani10071115 32610569 PMC7401522

[52] Hernández A Estrada-König D Romero-Zúñiga JJ Galina CS Berg C Rojas-Gonzales M. Implementation of the welfare quality protocol in dairy farms raised on extensive, semi-intensive and intensive systems in Costa Rica. J Anim Behav Biometeorol. (2017) 5:132–8. 10.31893/2318-1265jabb.v5n4p132-138

[53] Mancera KF Zarza H López de Buen L Carasco García AA Montiel Palacios F Galindo F. Integrating links between tree coverage and cattle welfare in silvopastoril systems evaluation. Agron Sust Develop. (2018) 38:19. 10.1007/s13593-018-0497-3

[54] Améndola L Solorio FJ Ku-Vera JC Améndola-Massioti RD Zarza H Mancera KF. A pilot study on the foraging behaviour of heifers in intensive silvopastoral and monoculture systems in the tropics. Animal. (2019) 3:606–16. 10.1017/S1751731118001532 29983122

[55] Deniz M Schmitt A Hötzel M de Sousa K Pinheiro L Sinisgalli P. Microclimate and pasture area preferences by dairy cows under high biodiversity silvopastoral system in Southern Brazil. Int J Biometeorol. (2020) 64:1877–87. 10.1007/s00484-020-01975-0 32737583

[56] Deniz M de Sousa KT Moro MF do Vale MM Dittrich JR Pinheiro Machado Filho LC. Social hierarchy influences dairy cows' use of shade in a silvopastoral system under intensive rotational grazing. Appl Anim Beh Sci. (2021) 244:105467. 10.1016/j.applanim.2021.105467

[57] Cartes D Strappini A Sepulveda-Varas P. Provision of shelter during the prepartum period: effects on behavior, blood analytes, and health status in dairy cows in winter. J Dairy Sci. (2021) 104:3508–21. 10.3168/jds.2020-19439 33455757

[58] Matamala F Strappini A Sepúlveda-Varas P. Dairy cow behaviour around calving: its relationship with management practices and environmental conditions. Aust J Vet Sci. (2021) 53:0719–8132. 10.4067/S0719-81322021000100009

[59] Gallo C Teuber C Cartes M Uribe H Grandin T. Mejoras en la insensibilización de bovinos con pistola neumática de proyectil retenido tras cambios de equipamiento y capacitación del personal. Arch Med Vet. (2003) 35:4. 10.4067/S0301-732X2003000200004

[60] Gallo C Lizondo G Knowles T. The effects of journey and lairage time on steers transported to slaughter in Chile. Vet Rec. (2003) 152:361–4. 10.1136/vr.152.12.361 12678260

[61] Gallo C Warriss P Knowles T Negrón R Valdés A Mencarini I. Densidades de carga utilizadas para el transporte de bovinos destinados a matadero en Chile. Arch Med Vet. (2005) 37:155–9. 10.4067/S0301-732X2005000200010

[62] Miranda-de la Lama G Leyva I Barrera-Serrano A Pérez-Linares C Sánchez-López E María G. Assessment of cattle welfare at a commercial slaughter plant in the northwest of México. Trop Anim Health Prod. (2012) 44:497–504. 10.1007/s11250-011-9925-y 21789548

[63] Miranda de la Lama G Gonzales C Gutiérrez F Villarroel M María G Estevez-Moreno LX. Welfare of horses from Mexico and the United States of America transported for slaughter in Mexico: fitness profiles for transport and pre-slaughter logistics. Prev Vet Med. (2020) 180:105033. 10.1016/j.prevetmed.2020.105033 32464300

[64] Huertas SM van Eerdenburg F Gil A Piaggio J. Prevalence of carcass bruises as an indication of welfare in beef cattle and the relation to the economic impact. Vet Med Sci. (2015) 1:9–15. 10.1002/vms3.2 29067169 PMC5645810

[65] Gallo C Pérez S Sanhueza C Gasic J. Efectos del tiempo de transporte de novillos previo al faenamiento sobre el comportamiento, pérdidas de peso y algunas características de la canal. Arch Med Vet. (2000) 32:157–70. 10.4067/S0301-732X2000000200003

[66] Gallo C Espinoza MA Gasic J. Efectos del transporte por camión durante 36 horas con y sin período de descanso sobre el peso vivo y algunos aspectos de calidad de carne en bovinos. Arch Med Vet. (2001) 3:43–53. 10.4067/S0301-732X2001000100005

[67] Gallo C. Using scientific evidence to inform public policy on the long distance transportation of animals in South America. Vet Ital. (2008) 44:113–20. Available online at: https://www.izs.it/vet_italiana/2008/44_1/113.pdf 20405418

[68] Huertas SM Gil A Piaggio JM van Eerdenburg FJCM. Transportation of Beef cattle to slaughterhouses and how this relates to animal welfare and carcase bruising in an extensive production system. Anim Welfare. (2010) 19:281–5.

[69] Romero M Sánchez V. Animal welfare during transport and its relationship with meat quality. Rev MVZ Cordoba. (2012) 17:2936–44. 10.21897/rmvz.26426967002

[70] Gallo C Taruman J Larrondo C. Main factors affecting animal welfare and meat quality in lambs for slaughter in Chile. Animals. (2018) 8:165. 10.3390/ani8100165 30262753 PMC6210305

[71] Tarazona A Ceballos M Broom D. Human relationships with domestic and other animals, one health, one welfare, one biology. Animals. (2019) 10:1–21. 10.3390/ani10010043 31878310 PMC7022888

[72] Bravo V Knowles T Gallo C. Transport, associated handling procedures and behaviour of calves marketed through Chilean auction markets. Animals. (2020) 10:2170. 10.3390/ani10112170 33233317 PMC7700271

[73] Sánchez M Bravo V Gallo C. Behavior and health indicators to assess cull cow's welfare in livestock markets. Front Vet Sci. (2020) 7:471. 10.3389/fvets.2020.00471 32851042 PMC7427490

[74] Ocampo A Cardozo A Tarazona A Ceballos M Murgueitio E. La investigación participativa en bienestar y comportamiento animal en el trópico de América: oportunidades para nuevo conocimiento aplicado. Rev Colomb Cs Pec. (2011) 24:332–46. Available online at: http://www.scielo.org.co/pdf/rccp/v24n3/v24n3a14.pdf

[75] Schnaider MA Heidemann MS Silva AHP Taconeli CA Molento CFM. Vocalization and other behaviors indicating pain in beef calves during the ear tagging procedure. J Vet Behav Clin Appl Res. (2022) 47:93–8. 10.1016/j.jveb.2021.10.005

[76] Ceballos MC Sant'Anna AC Boivin X de Oliveira Costa F Carvalhal M Paranhos da Costa M. Impact of good practices of handling training on beef cattle welfare and stockpeople attitudes and behaviors. Livest Sci. (2018) 216:24–31. 10.1016/j.livsci.2018.06.019

[77] Cedeño-Palacios C Delgado M Duenas A Alcivar U Vasquez L. Compliance with good livestock practices in selected farms in Ecuador. Rev Ci-Facultad de Ciencias Veterinarias. (2019) 29:101–6. Availabler online at: https://produccioncientificaluz.org/index.php/cientifica/article/view/29592/30377

[78] Mendes P Siqueira H Siqueira A Prata L. Diagnosis of compliance with animal welfare standards in broiler slaughterhouse located in the state of Goiás. PUBVET. (2019) 3:5. 10.31533/pubvet.v13n5a325.1-7

[79] Mamani-Linares L Gallo C. A note on the effects of pre-slaughter operations of llamas (*Lama glama*) on the concentrations of some blood constituents related to stress and carcass quality. Arch Med Vet. (2014) 46:463–9. 10.4067/S0301-732X2014000300018

[80] Smith C Mendoza G Gustavo C Ghezzi M. Evaluación de las condiciones de bienestar animal de camélidos sudamericanos ingresados al camal municipal de Huancavelica, Perú. Rev Mexicana Cs Pec. (2019) 10:379–90. 10.22319/rmcp.v10i2.4568

[81] Guerrero Pincay AE González Marcillo RL Castro Guamàn WE Ortiz Naveda NR Grefa Reascos DA Guamàn Rivera SA. Influence of litter size at birth on productive parameters in guinea pigs (*Cavia porcellus*). Animals. (2020) 10:2059. 10.3390/ani10112059 33171794 PMC7695018

[82] Larrondo C Orihuela A Strappini A Acosta G Mota-Rojas D Gallo C. Provision of straw and presence of undocked lambs reduce the behavioral and physiological expressions of pain and stress associated with tail docking in lambs: a preliminary study. Anim Prod Sci. (2020) 621:423–31. 10.1071/AN20237

[83] Hoetzel MJ Sneddon JN. The role of extensionists in Santa Catarina, Brazil, in the adoption and rejection of providing pain relief to calves. J Dairy Sci. (2013) 96:1535–48. 10.3168/jds.2012-5780 23332854

[84] Larrondo C Bustamante H Gallo C. Sheep farmers perception of welfare and pain associated to routine husbandry practices in Chile. Animals. (2018) 8:225. 10.3390/ani8120225 30487400 PMC6315487

[85] Larrondo C Bustamante H Paredes E Gallo C. Long term hyperalgesia and traumatic neuroma formation in tail docked lambs. Anim Welfare. (2019) 28:443–54. 10.7120/09627286.28.4.443

[86] Herzberg D Strobel P Chihuailaf R Ramírez-Reveco A Müller H Werner M. Spinal reactive oxygen species and oxidative damage mediate chronic pain in lame dairy cows. Animals. (2019) 9:693. 10.3390/ani9090693 31533257 PMC6770087

[87] Andrighetto M Rossi J Jardim J. Attitudes of cattle veterinarians and animal scientists to pain and painful procedures in Brazil. Prev Vet Med. (2020) 177:104909. 10.1016/j.prevetmed.2020.104909 32145531

[88] Hoetzel MJ Yunes MC Vandresen B Albernaz-Goncalves R Woodroffe RE. On the road to end pig pain: knowledge and attitudes of Brazilian citizens regarding castration. Animals. (2020) 10:1826. 10.3390/ani10101826 33049950 PMC7650544

[89] Stampa E Schipmann C Hamrn U. Consumer perceptions, preferences, and behavior regarding pasture-raised livestock products: a review. Food Qual Prefer. (2020) 82:103872. 10.1016/j.foodqual.2020.103872

[90] Schnettler B Vidal R Silva R Vallejos L Sepúlveda N. Consumer perception of animal welfare and livestock production in the Araucania Region, Chile. Chilean J Agric Res. (2008) 68:80–93. 10.4067/S0718-58392008000100008

[91] Schnettler B Vidal R Silva R Vallejos L Sepúlveda N. Consumer willingness to pay for beef meat in a developing country: the effect of information regarding country of origin, price and animal handling prior to slaughter. Food Qual Pref. (2009) 20:156–65. 10.1016/j.foodqual.2008.07.006

[92] Villalobos P Padilla C Ponce C Rojas A. Beef consumer preferences in Chile: importance of quality attribute differentiators on the purchase decision. Chilean J Agric Res. (2010) 70:85–94. 10.4067/S0718-58392010000100009

[93] Vargas-Bello-Pérez E Miranda-de la Lama G Lemos Teixeira D Enríquez-Hidalgo D Tadich T Lensink J. Farm animal welfare influences on markets and consumer attitudes in Latin America: the cases of Mexico, Chile and Brazil. J Agric Environ Ethics. (2017) 30:697–713. 10.1007/s10806-017-9695-2

[94] Vargas-Bello E Obermöller C Faber I Tadich T Toro P. Knowledge and perception on animal welfare in Chilean undergraduate students with emphasis on dairy cattle. Animals. (2021) 11:1921. 10.3390/ani11071921 34203442 PMC8300360

[95] Teixeira D Larraín R Hötzel M. Are views towards egg farming associated with Brazilian and Chilean egg consumers' purchasing habits? PLoS ONE. (2018) 13:e0203867. 10.1371/journal.pone.0203867 30265672 PMC6161848

[96] Cornish A Briley D Wilson B Raubenheimer D. The price of good welfare: does informing consumers about what on-package labels mean for animal welfare influence their purchase intentions? Appetite. (2020) 148:104577. 10.1016/j.appet.2019.104577 31904389

[97] Sinclair M Zhang Y Descovich K Phillips CJC. Farm animal welfare science in China—a bibliometric review of Chinese literature. Animals. (2020) 10:540. 10.3390/ani10030540 32213957 PMC7142485

[98] Huertas SM Bobadilla PE Alcántara I Akkermans E van Eerdenburg FJCM. Benefits of silvopastoral systems for keeping beef cattle. Animals. (2021) 11:992. 10.3390/ani11040992 33916155 PMC8066609

[99] Broom DM Galindo FA Murgueitio E. Sustainable, efficient livestock production with high biodiversity and good welfare for animals. Proc R Soc B Biol Sci. (2013) 280:1771. 10.1098/rspb.2013.2025 24068362 PMC3790492

[100] Tadich N Gallo C Brito M Broom D. Effect of weaning and 48 h transport by road and ferry on some blood indicators of welfare in lambs. Livestock Sci. (2009)121:132–6. 10.1016/j.livsci.2008.06.001

[101] Strappini A Frankena K Metz J Gallo C Kemp B. Characteristics of bruises in carcasses of cows sourced from farms or from livestock markets. Animal. (2012) 6:502–9. 10.1017/S1751731111001698 22436230

[102] Ungerfeld R Hötzel M Quintans G. Changes in behaviour, milk production and bodyweight in beef cows subjected to two-step or abrupt weaning. Anim Prod Sci. (2015) 55:1281–8. 10.1071/AN13453

[103] Galindo F de Aluja A Cagigas R Huerta LA Tadich T. Application of the hands-on donkey tool for assessing the welfare of working equids at Tuliman, Mexico. J Appl Anim Welf Sci. (2018) 21:1. 10.1080/10888705.2017.1351365 28762781

[104] Hötzel M Mota S Ludtke C Poletto R. Knowledge and attitudes of official inspectors at slaughterhouses in southern Brazil regarding animal welfare. Rev Brasil Zootech Brazil J Anim Sci. (2018) 47:65. 10.1590/rbz4720170065

[105] Freitas-de-Melo A Ungerfeld R Orihuela A Hötzel MJ Pérez-Clariget R. Early mother–young relationship and feeding behaviour of lambs are unaffected by low pasture allowance until the beginning of the last third of gestation in single-bearing ewes. Anim Prod Sci. (2017) 58: 930–6. 10.1071/AN16157

